# Agricultural by-products and oyster shell as alternative nutrient sources for microbial sealing of early age cracks in mortar

**DOI:** 10.1186/s13568-020-01166-5

**Published:** 2021-01-06

**Authors:** Minyoung Hong, Indong Jang, Yongjun Son, Chongku Yi, Woojun Park

**Affiliations:** 1 Laboratory of Molecular Environmental Microbiology, Department of Environmental Science and Ecological Engineering, Korea University, Seoul, 02841 Republic of Korea; 2 Construction Management and Material Laboratory, School of Civil, Environmental and Architectural Engineering, Korea University, Seoul, 02841 Republic of Korea

**Keywords:** Bio-concrete, Economical medium, Calcium source, Calcium carbonate precipitation, Acid treatment, Bacterial spore

## Abstract

Bio-concrete using bacterially produced calcium carbonate can repair microcracks but is still relatively expensive due to the addition of bacteria, nutrients, and calcium sources. Agricultural by-products and oyster shells were used to produce economical bio-concrete. Sesame meal was the optimal agricultural by-product for low-cost spore production of the alkaliphilic *Bacillus miscanthi* strain AK13. Transcriptomic dataset was utilized to compare the gene expressions of AK13 strain under neutral and alkaline conditions, which suggested that NaCl and riboflavin could be chosen as growth-promoting factors at alkaline pH. The optimal levels of sesame meal, NaCl, and riboflavin were induced with the central composite design to create an economical medium, in which AK13 strain formed more spores with less price than in commercial sporulation medium. Calcium nitrate obtained from nitric acid treatment of oyster shell powder increased the initial compressive strength of cement mortar. Non-ureolytic calcium carbonate precipitation by AK13 using oyster shell-derived calcium ions was verified by energy-dispersive X-ray spectroscopy and X-ray diffraction analysis. Stereomicroscope and field emission scanning electron microscopy confirmed that oyster shell-derived calcium ions, along with soybean meal-solution, increased the bacterial survival and calcium carbonate precipitation inside mortar cracks. These data suggest the possibility of commercializing bacterial self-healing concrete with economical substitutes for culture medium, growth nutrient, and calcium sources.

## Key points

- Sesame meal and soybean meal were used for bacterial growth and sporulation.
- Oyster shells were treated with nitric acid to extract calcium ions.
- Increased bacterial survival and crack-sealing were observed by the oyster-calcium ions.

## Introduction

Bio-concrete is a bacteria-based self-healing concrete that can fill cracks without human intervention (de Rooij et al. 2013). Concrete structures inevitably crack under the pressure of tensile stress caused by plastic shrinkage, tensile load, and a wide range of chemical reactions (Mehta and Monteiro 2014). External pollutants such as carbonate, chloride ion, and sulfate penetrate concrete when cracks occur, causing concrete deterioration and corrosion of steel reinforcement (Jonkers and Schlangen 2007; Zhang et al. 2017). Bio-concrete can self-seal cracks through microbially-induced calcium carbonate precipitation (MICP), in which calcium carbonate is formed by bacterial metabolic activities and prevent penetration of external contaminants in advance (Jonkers et al. 2010; Vijay et al. 2017; Lee and Park 2018). Bacteria commonly used to develop self-healing concrete, e.g. *Bacillus cohnii*, *Lysinibacillus sphaericus*, and *Sporosarcina pasteurii*, are Gram-positive bacteria with a negatively-charged cell wall capable of binding to surrounding cations such as calcium ions (Zhang et al. 2015; Seifan et al. 2016; Vijay et al. 2017). The cell wall-bound calcium ions then bind to bacterially-produced carbonate and form a calcium carbonate precipitate, which fills cracks in the concrete (Jonkers et al. 2010; De Belie and Wang 2015). Bio-concrete has economic and environmental significance. Self-healing concrete reduces labor costs for crack repair, prevents the spread of cracks early to improve structure durability, and reduces the carbon footprint of concrete production and transport (Jonkers et al. 2010). In addition, the calcium-carbonate-precipitation-based bacterial self-healing of concrete is more environmentally friendly than other inorganic admixtures and has excellent compatibility with cementitious materials (Seifan et al. 2016). However, the high production cost of bio-concrete should be reduced for its complete commercialization.

Three factors are required to produce bacterial self-healing concrete: carbonate-producing bacteria, nutrients for their growth and metabolism, and a calcium source to promote calcium carbonate precipitation (Jonkers et al. 2010). However, the production cost of bio-concrete is as much as 50% higher per cubic meter than concrete due to the need to cultivate of target bacteria and addition of nutrients and calcium substrate. This cost differential threatens to undermine the advantages of concrete as an inexpensive building material (Lee and Park 2018; Peplow 2020). The price of the self-healing agents applied per m^3^ of concrete should be reduced by €15 to €20 (Silva et al. 2015). One method to reduce the initial cost of bio-concrete is bacterial cultivation in a low-cost media. Cultivation can account for up to 60% of the total bacterial production cost, and 50% of the final-product price can be determined by the price of raw materials (Makkar and Cameotra 1999; Kristiansen 2001). The use of inexpensive agricultural by-products for bacterial culture could lower the production cost of bio-concrete. The nutritional content of agricultural by-products (e.g., carbon, nitrogen and minerals) give them great potential as culture media for bioengineering processes that might be more effective than commercial media and cost less (Sasaki et al. 1998; Heck et al. 2002; Chen et al. 2010; Salihu et al. 2012).

Oyster shell waste contains abundant calcium ions (37.4% of dry weight) making it an economical calcium source for bio-concrete substrate (Kwon et al. 2004). Up to 96% of oyster-shell components are composed of calcium carbonate, which can be treated with acidic solutions to release the calcium ions (Yoon et al. 2003). Oyster is a major seafood and is cultivated in shellfish farms that cover a total of 4000 ha on the southern coast of Korea (KISEI 2011). About 32,000 tons of oyster flesh are produced per year due to the optimal conditions of high water temperatures, low depth, and calm water due to the development of complex coastlines with bays of the southern coast (KMI 2018). However, only 10 percent of the 280,000 tons of oyster shells that are generated every year are reused as fertilizers or industrial raw materials led by the government due to insufficient management of processing facilities and limitations in treatment capacity. The remaining untreated oyster-shell waste is left on the coast, causing community disruptions such as malodor or landscape damage (KISEI 2011). Oyster shells have been mixed with concrete as a building material to solve the environmental problems, but there are no precedents for its use in bio-concrete (Yoon et al. 2003; Yang et al. 2005, 2010).

The purpose of this study is to explore the possibility of using agricultural by-products and oyster shells as economical substitutes for nutrients for bacterial culture, metabolic activities, as well as calcium source which required to manufacture bio-concrete. A transcriptomic dataset which represents the comparison of gene expression during growth at pH 7 and 10 was used to select factors that support the growth of AK13 under alkaline conditions. The oyster shell was treated with nitric acid to separate calcium ions, which were then mixed with cement mortar to verify their effect on the compressive strength and crack-sealing.

## Materials and methods

### Preparation of seed culture and culture conditions

The calcium-carbonate precipitating bacterium, *Bacillus miscanthi* AK13 strain (available as KACC 21401^T^, DSM 109981^T^) used to produce bacteria-added mortar specimens was isolated from the rhizosphere of *Miscanthus sacchariflorus* in Seongbukcheon, Seoul, Korea. The AK13 strain is an alkaliphilic bacterium capable of forming colonies at pH 12 (Lee and Park 2019). The AK13 seed culture was prepared using Luria–Bertani (LB) medium (5 g/L of yeast extract, 10 g/L of tryptone, and 10 g/L of NaCl) and incubated for 15 h before inoculation. In all cases, the AK13 cells were washed with phosphate buffered saline (PBS) solution and then standardized to an OD_600_ value of 0.5 using a multimode microplate reader (Spark, Tecan, Switzerland), prior to a 1% (v/v) inoculation into experimental media. Cultures were incubated at 30 ℃ with shaking at 220 rpm. All media were adjusted to pH 10 with Na_2_CO_3_ unless otherwise specified. All reagents used in the experiment were guaranteed reagent grade.

### Selection of agricultural by-products

All agricultural by-products, i.e. perilla meal, rice bran, sesame meal, soybean meal, soybean pulp, and wheat bran, were dried at 50 °C then ground with a blender to homogenize the particle size. Each by-product was mixed with 50 mL of distilled water at 10 g/L in a 250 mL Erlenmeyer flask then sterilized at 121 °C for 20 min. After sterilization, AK13 was inoculated into each by-product-media and cultured with shaking for 72 h. Serial dilution was used to measure AK13 growth whereby each 72 h culture was serially diluted in PBS from 10^–1^ to 10^–6^, then 20 µL spotted onto pH 8 LB plates with at least 2 repetitions. To count the number of spores, the culture media was incubated at 80 °C for 15 min to kill vegetative cells and serially diluted as described above. The by-product cultures followed by cell and spore counts were performed in triplicate. Difco™ sporulation (DS) medium, a commercial sporulation medium, consisted of 8 g/L nutrient broth (BD), 1 g/L KCl, and 0.25 g/L MgSO_4_ with filter-sterilized 164 mg/L Ca(NO_3_)_2_, 1.26 mg/L MnCl_2_, and 0.15 mg/L FeSO_4_.

### RNA sequencing and growth-promoting factor screening

AK13 cells were grown to the mid-exponential phase in both pH 7 and pH 10 LB medium buffered with NaH_2_PO_4_–Na_2_HPO_4_ and Na_2_CO_3_, respectively (Additional file 1: Fig. S1a). Total RNA was obtained using a RNeasy kit (Qiagen, Hilden, Germany) from mid-exponentially grown AK13 cells from pH 7 and 10 LB media. RiboZero rRNA removal kit (Epicentre, Medison, USA) was used to delete ribosomal RNA from isolated total RNA. Libraries for Illumina sequencing were made with the Universal library (Tecan, USA) following the manufacturers introductions. Illumina HiSeq 2500 platform performed paired-end 100 bp RNA sequencing. The reference genome (*Bacillus miscanthi* AK13) to match sequence data was retrieved from the NCBI database. Bowtie2 aligned quality-filtered reads to the reference genome sequence. The abundance of relative transcript was shown by reads per kilobase of the exon sequence per million mapped sequence reads (RPKM) defined as total exon reads/(mapped reads in millions exon length in kilobases). CLRNASeq program (Chunlab, Korea) was used to analyzed Metabolic pathways based on KEGG pathway and BLAST alignment with proteins. Raw RNA sequencing data were deposited into the Sequence Read Archive (SRA) of the National Center for Biotechnology Information (NCBI), accession number SRX8695687.

Riboflavin, thiamine, biotin, and tryptophan were each added to pH 7 and pH 10 LB media at concentrations between 10^0^ and 10^–6^ g/L at one log intervals. The growth of AK13 was monitored by OD_600_ every hour for up to 12 h. The maximum growth rate of strain AK13 in each substrate was calculated to identify the supplement and concentration that yielded the highest growth-promoting effects. Each measurement was performed in triplicate.

### Nutritional factor optimization

A central composite design of response surface methodology was used to optimize three nutritional factors (Additional file 1: Tables S1 and S2).We set five levels for the nutritional factors and prepared a design table for optimization with MINITAB (ver. 14; Minitab Inc., USA). Experimental media were prepared by combining the three nutritional factors as suggested by the design table, inoculated with AK13, and incubated for 72 h.

### Separation of calcium ions through acid treatment on oyster shells

Varying amounts of powdered oyster shells (0, 1, 3, 5, 10, 15, 20, and 25 g) were added to 100 mL of 1 M nitric acid solution and shaken for 8 h. Each solution was filtered through a 5 µm Whatman® filter paper (Sigma-Aldrich, USA) to remove any remaining solids prior to pH measurement with an Orion Versa Star Pro electrode (Thermo Fisher Scientific, USA). To prepare B4 medium modified with oyster shell-derived calcium ions or B4O medium, 0.4 g of the powdered oyster shell was mixed with 100 mL of 0.1 M nitric acid solution, and after shaking for 8 h, and impurities were filtered out with a Whatman® filter paper. The passed solution was mixed with 0.4 g of yeast extract to form a B4O medium and then autoclaved.

### Collection and verification of microbially precipitated calcium carbonate

Sterilized B4O medium was inoculated with AK13 after adjusting the pH to 10 with filter-sterilized 2 M NaOH and cultured for 96 h. Calcium carbonate precipitate was extracted by heat-lysing the cells at 80 °C for 10 min followed by one minute in the microwave and centrifugation at 7830 rpm for 10 min. The amount of precipitate collected was quantified using an electronic scale. Three experimental replicates were performed. Field emission scanning electron microscopy (FE-SEM), energy-dispersive X-ray (EDX) spectroscopy, and X-ray diffraction (XRD) analysis were performed to verify the calcium carbonate precipitated in the B4O medium. AK13 cells and precipitates from B4O medium were firstly fixed for 2 h in low-strength Karnovsky’s solution (2% paraformaldehyde, 2.5% glutaraldehyde, and 0.1 M phosphate buffer, final pH 7.2) and then for 2 h in 2% osmium tetroxide solution. These fixed samples were gradually dehydrated with ethanol (30%, 50%, 70%, 100%) for 10 min each and placed on an aluminum stub to be dried at room temperature for 2 days. These samples were coated with platinum before FE-SEM/EDX analyses (Quanta 250 FEG, FEI, USA). XRD analysis was performed using SmartLab (Rigaku, Japan) on lyophilized powder of AK13 cells and precipitates obtained from B4O medium.

### Mortar preparation and compressive strength measurement

Mortar specimens were prepared based on ASTM C349, a standard test method for the compressive strength of hydraulic-cement mortars. Type I Portland cement and ISO standard sand (ISO 679:2009) were mixed to manufacture four types of mortar (Table 1): reference mortar (Ref), oyster shell-derived calcium ion-inclusive mortar (Oys), mortar supplemented with AK13 spores (Spo), and mortar supplemented with both calcium ions and spores (Mix). All mortar mixtures were cast in a 40 mm × 40 mm × 160 mm mold for compressive strength measurements, a 40 mm × 40 mm × 10 mm mold for crack-sealing experiments, and then cured at room temperature (23 °C) for 24 h before demolding. Small-sized mortar for crack-sealing experiments were prepared to facilitate microscopic observation of the specimens. Mortar specimens were submerged in tab water at room temperature until testing. Compressive strength was measured on 40 × 40 × 160 mm prismatic specimens according to the ASTM C349 after 3, 7, and 28 days of water curing. Six samples were created for the measurement of each mortar type, and the average compressive strength and standard deviation was calculated.

**Table 1:** The mortar specimen mixtures

Type	Cement (g)	Sand (g)	Water (g)	Calcium solution (g)	Spore pellet (g)^a^
Ref	1350.0	4050.0	625.0	0	0
Oys	1350.0	4050.0	312.5	312.5	0
Spo	1350.0	4050.0	620.5	0	4.5
Mix	1350.0	4050.0	308.0	312.5	4.5

^a^2.23 × 10^10^ CFU/g

### Preparation of nutrient solution for AK13 growth and germination

Six agricultural by-products were each added to distilled water at a concentration of 10 g/L, autoclaved, and then centrifuged at 7830 rpm for 20 min to clarify the supernatant. The supernatants were then adjusted to pH 10 with Na_2_CO_3_, inoculated with AK13 and cultured with shaking at 30 ℃. At 12 and 24 h post-inoculation, cell counts were measured by serial dilution to identify the substrate with the highest growth rate of AK13. To identify the substrate with the maximum germination rate for AK13 spores, pH 10 and 1.5% agar plates were made with clarified supernatant from each agricultural by-product. Purified spores were serially diluted in distilled water from 10^–1^ to 10^–5^ and 10 µL aliquots spotted onto each plate. Spores were purified using lysozyme and sodium dodecyl sulfate (Ryu et al. 2020).

### Confirmation of crack-sealing of cement mortar

After 7 days of water curing, the 40 mm × 40 mm × 10 mm prismatic specimens were split into two pieces to create cracks. The pieces of the specimens were reattached and fixed by wrapping a parafilm around the edge, and the initial crack-widths were set to 0.3 mm by inserting a silicon sheet having a constant thickness between the cracks. All crack-induced specimens were placed on petri dishes and maintained half immersed in tap water throughout the sealing period. Twelve lines were drawn over the crack per specimen for measurement of the crack width. Nutrient solutions made from selected agricultural by-product were administered in 1 mL aliquots to the cracks daily until completely sealed (up to 14 days). Crack images were taken 0, 3, 7, and 14 days after crack induction using a Colony Doc-It Imaging station (Analytik Jena AG, Germany) and a microscope (ImagerA1, Zeiss, Germany). The crack widths were measured at line-drawn points with ImageJ software (National Institutes of Health, USA). A stereomicroscope (SZX7, Olympus, Japan) was used to compare the calcium carbonate precipitation in the cracks on day 14. The calcium carbonate precipitates were then scraped into a 1.5 mL microcentrifuge tube containing 95% ethanol using sterilized pipette tips. The suspended precipitate was then placed on an aluminum stub and dried for FE-SEM imaging. Three specimens of each mortar type were prepared to confirm crack-sealing and ensure the accuracy of the results.

### Statistical analysis

The statistical significance between means was determined by Fisher’s *F* test with Excel (Microsoft, USA) and MINITAB (ver. 14). The Student’s *t* test was used to determine significance of the regression model.

## Results

### Selection of agricultural by-product and growth-promoting factors

**Fig. 1:**
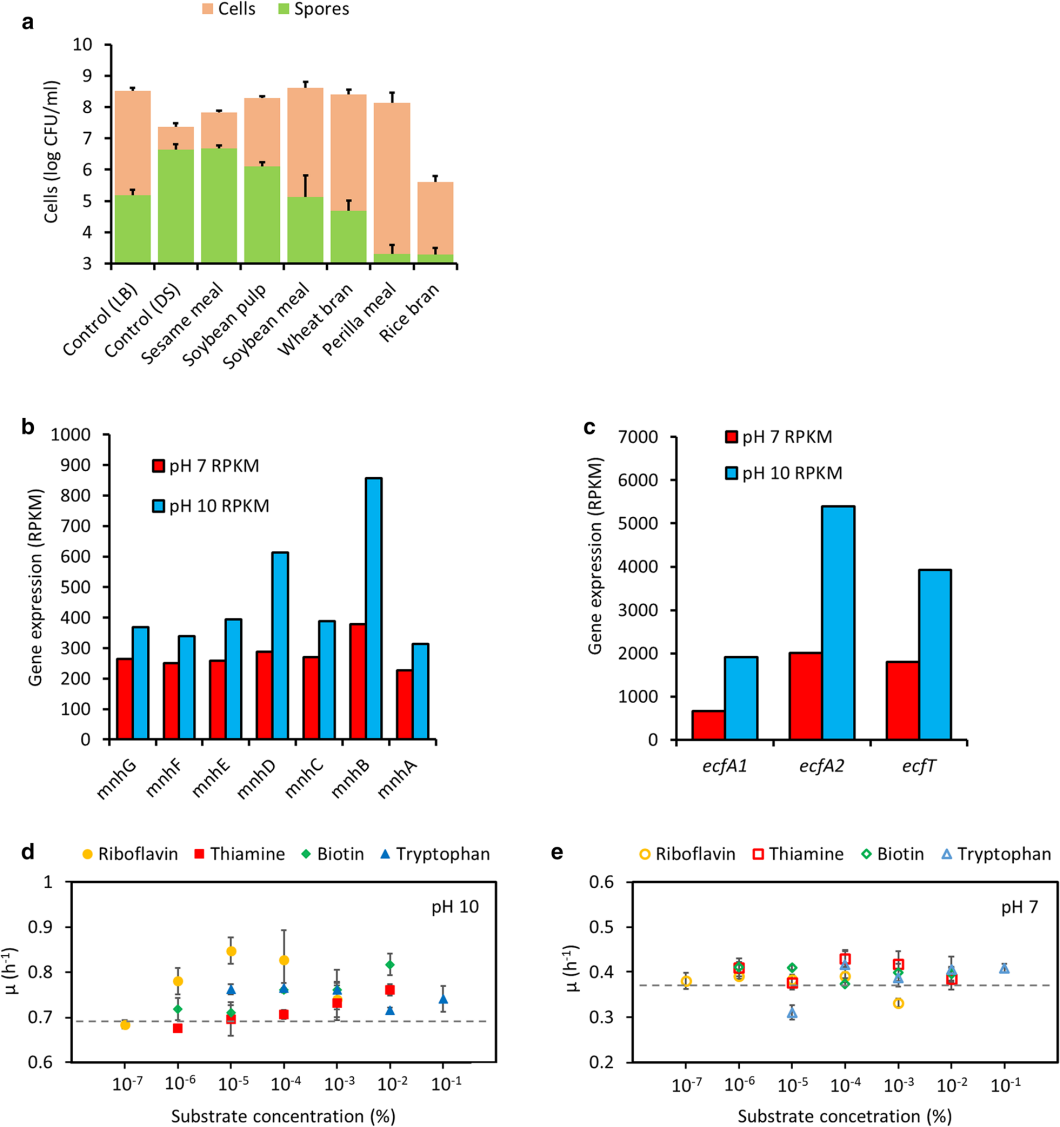
Selection of agricultural by-product to make an economical medium for the growth and sporulation of AK13. **a** Selection of most effective agricultural by-product for AK13 spore formation. **b** Comparison of gene expression in Mrp antiporter operon at pH 7 and 10. **c** Expression of genes related to ECF transporter in pH 7 and 10 growth conditions. **d**, **e** Changes in the growth rate of AK13 following supplementation with four ECF transporter substrates in pH 7 and 10 LB media. The dotted lines indicate the growth rates of AK13 without supplementation

To take advantage of spore durability, we selected *B. miscanthi* strain AK13 and conducted a screen of six agricultural by-products to identify a substrate that maximizes spore formation. Each by-product was used at a concentration of 10 g/L (see “Materials and methods”), The highest amount of spores (4.8 ± 0.1 × 10^6^ spores/mL) in sesame meal (SM), which was similar level of spores yields (4.7 ± 0.2 × 10^6^ spores/mL) in commercial DS medium (Fig. 1a). Total cell counts in SM-medium were also measured higher than those in the DS medium, so SM was selected as a spore-forming substrate for AK13. Less amount of AK13 spores were induced in the other agricultural by-products except SM compared to commercial media. Perilla meal and rice bran recorded the lowest spore formation. Compared with SM, soybean pulp, soybean meal, wheat bran, and perilla meal recorded higher total cell counts, but only 0.1–27.2% of viable spores were measured.

Transcriptomic data were collected from AK13 cells grown in pH 7 and pH 10 LB media and analyzed to select factors that promote AK13 growth in alkaline conditions like culture media or concrete matrix. The number of genes up-regulated at pH 10 was eight times greater than those at pH 7 (Additional file 1: Fig. S1b). The expression of Mrp antiporter-related operon (*mnhA-G*) were up-regulated at pH 10 (Fig. 1b and Table 2). The Mrp antiporter excretes sodium ions and uptakes hydrogen ions to help maintain the pH homeostasis of alkaliphilic *Bacillus* spp. in environments with high alkalinity (Ito et al. 2017). Therefore, NaCl was selected as a growth-promoting factor supporting SM to supply sodium ions into the SM-medium. Energy-coupling factor (ECF) transporter-related genes were also included among the seventeen genes belonging to the transport and metabolism of nutrients (e.g. carbohydrate, inorganic ion, amino acid, etc.) up-regulated under alkaline conditions (Fig. 1c and Table 2). In the alkaline condition, the expressions of ECF transporter-forming *ecfA1*, *ecfA2*, and *ecfT* were also more than doubled compared to the neutral condition. A screen of these four micronutrients (see “Materials and methods”) identified riboflavin as the most effective growth-promoting nutrient for AK13 at pH 10. Supplementation of pH 10 LB medium with 1 × 10^–5^% riboflavin recorded the highest increase in AK13 growth (Fig. 1d) but not at pH 7 (Fig. 1e). The growth rates of AK13 improved with NaCl and riboflavin supplementation of SM-medium (Additional file 1: Fig. S3a), but there was no significant difference in the amount of spore formation (Additional file 1: Fig. S3b).

**Table 2:** Genes belonging to the transport and metabolism COG category whose expression increased by at least 1.5 times in alkaline conditions (RPKM > 100, *p* < 0.05)

Locus tag	Gene	Product	pH 7 RPKM	pH 10 RPKM	Fold change (pH 10/pH 7)
Genes related to carbohydrate transport and metabolism					
FPV17_RS05560	*ywqE*	Tyrosine-protein phosphatase	436.38	245.44	0.56
FPV17_RS14580	*ptsP*	Phosphoenolpyruvate-protein phosphotransferase	433.77	855.22	1.97
FPV17_RS14585	*ptgsH*	Phosphocarrier protein HPr	383.75	651.25	1.7
FPV17_RS14895	*pfkA*	ATP-dependent 6-phosphofructokinase	369.30	632.99	1.71
FPV17_RS15935		2,3-Bisphosphoglycerate-independent phosphoglycerate mutase	1653.74	3078.62	1.86
FPV17_RS15940		Triosephosphate isomerase	1799.09	3587.24	1.99
FPV17_RS15945		Phosphoglycerate kinase	503.95	991.99	1.97
FPV17_RS15950	*gap*	Glyceraldehyde-3-phosphate dehydrogenase 1	995.56	1758.46	1.77
Genes related to inorganic ion transport and metabolism					
FPV17_RS00985	*ecfA1*	Energy-coupling factor transporter ATP-binding protein	671.65	1915.06	2.85
FPV17_RS00990	*ecfA2*	Energy-coupling factor transporter ATP-binding protein	2009.30	5400.02	2.69
FPV17_RS00995	*ecfT*	Energy-coupling factor transporter transmembrane protein	1798.58	3928.33	2.18
FPV17_RS08485	*mnhE*	Na(+)/H(+) antiporter subunit E	258.53	395.44	1.53
FPV17_RS08490	*mnhD*	Na(+)/H(+) antiporter subunit D	289.14	613.91	2.12
FPV17_RS08500	*mnhB*	Na(+)/H(+) antiporter subunit B	378.13	858.10	2.27
Genes related to inorganic ion transport and metabolism					
FPV17_RS17765	*ald*	Alanine dehydrogenase	283.32	712.21	2.51
FPV17_RS15165		Putative aminopeptidase YtoP	618.30	931.28	1.51
Genes related to nucleotide transport and metabolism					
FPV17_RS00945		Adenylate kinase	647.51	1108.33	1.71
FPV17_RS12920		Dihydroorotase	287.41	433.64	1.51
Genes related to lipid transport and metabolism					
FPV17_RS12755	*acpP*	Acyl carrier protein	299.89	549.18	1.83

### Optimization of nutrition factor followed by measurement of growth curves

**Fig. 2:**
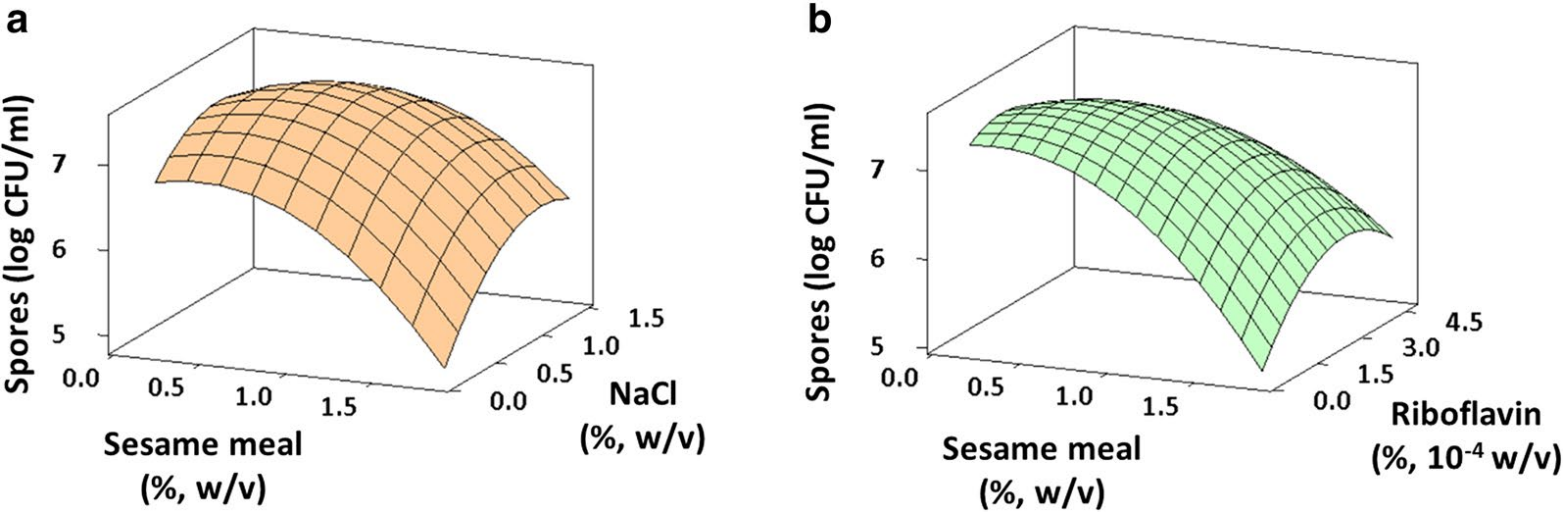
Interactions between growth factors visualized as a three-dimensional response surface plot. Interaction between **a** sesame meal and NaCl, **b** sesame meal and riboflavin. Interaction between NaCl and riboflavin was not plotted due to the lack of statistical significance

**Fig. 3:**
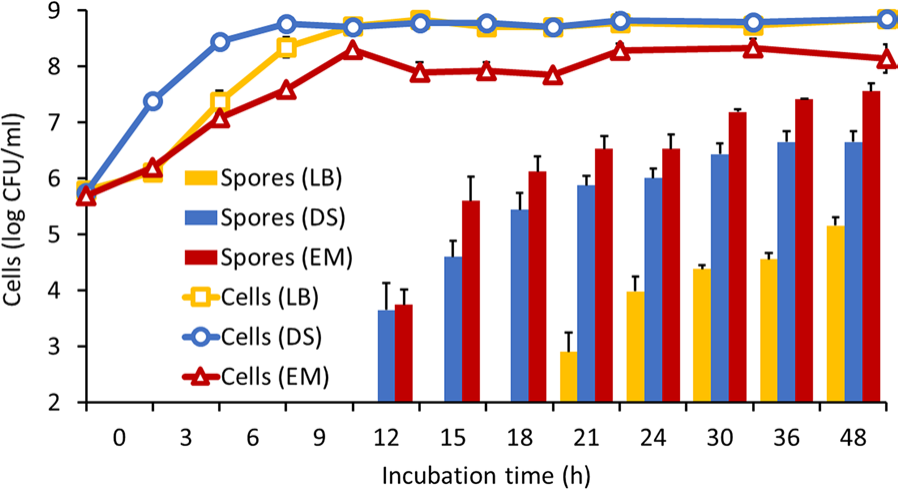
Growth and sporulation kinetics in EM and commercial media. The line graph showing the change in the total number of cells. Error bars represent standard deviation obtained through triplicate experiments. Error bars smaller than the mark are not displayed

The central composite design was used to determine the optimal levels of three selected nutritional factors, i.e. SM, NaCl, and riboflavin to maximize AK13 sporulation. Multiple regression analysis was performed based on the amount of AK13 spores formed in the experimental media compositions detailed by the design table (Additional file 1: Tables S1 and S2). The result was as follows:

$${\text{Y}} = 6.49 + 0.09X_{1} + 1.74X_{2} + 0.29X_{3} - 0.93X_{1}^{2} - 1.13X_{2}^{2} - 0.12X_{3}^{2} + 0.50X_{1} X_{2} + 0.25X_{1} X_{3} + \varepsilon .$$

where Y refers to the number of spores formed by AK13 when each variable (SM, NaCl, riboflavin) was at the indicated value. The ε represents the residual, i.e. the difference between the observed value and that predicted by the regression equation. According to the *p*-values obtained from Student’s *t* test, the term *X*_2_*X*_3_ was not statistically significant (*p* > 0.05), so it was pooled for accuracy and simplicity of the model. All other values, excepting *X*_1_, was significant at either *p* < 0.05 or *p* < 0.001. The *p*-value of Fisher’s *F* test from the analysis of variance (ANOVA) was significant (p < 0.001) at the 95% confidence level for all terms including the interactions. The coefficient of determination values (R^2^) were acceptable (R^2^ = 91.6%; adj. R^2^ = 90.2%), which means that the regression model fit the data sufficiently. The relationships between each factor were represented by surface plots to determine the maximum response, i.e. the spore yields of strain AK13 (Fig. 2a, b). The condition at which AK13 maximizes spore formation is at the ridges of the surface plots. The optimal levels of SM, NaCl, and riboflavin were determined to be 0.62 g, 0.93 g, and 1.87 × 10^–4^ g per 100 mL, respectively. Strain AK13 produced up to 7.9 times more spores at 48 h in the economical medium (EM) composed of optimized growth factors compared to the synthetic sporulation medium (Fig. 3).

### FE-SEM, EDX, and XRD analysis of calcium carbonate precipitants in B4O medium

**Fig. 4:**
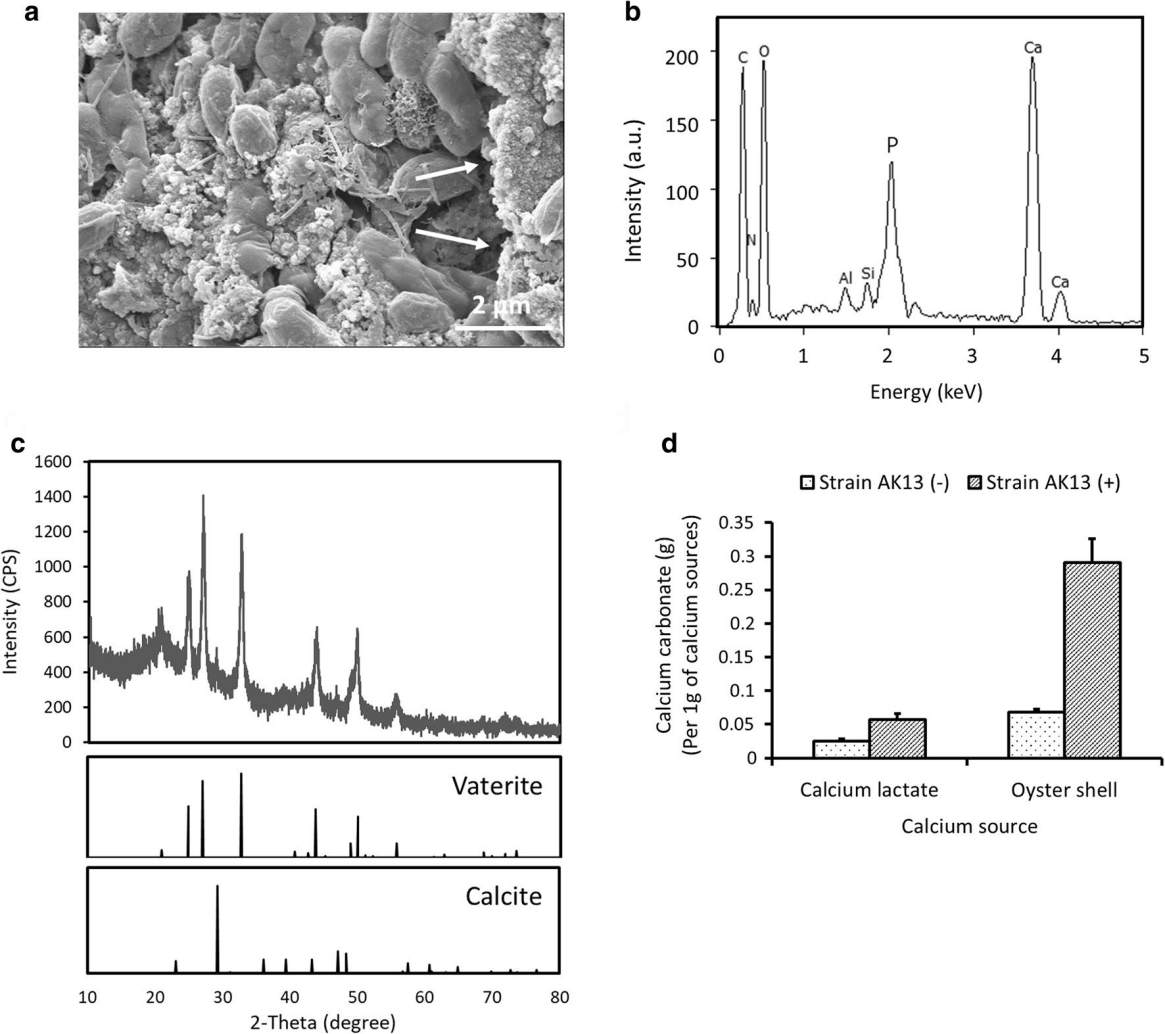
Calcium carbonate precipitated using oyster shell-derived calcium ions by AK13. **a** FE-SEM image of calcium carbonate precipitated by AK13 growth in B4O medium. **b**, **c** EDX and XRD analysis of calcium carbonate precipitated in B4O medium. **d** Comparison of the amount of calcium carbonate precipitated by AK13 with the calcium ions separated from the same mass of calcium lactate and oyster shell powder

The optimal amount of oyster shell powder was determined for calcium ion release by nitric acid. The addition of 25 g of powdered oyster shells to 100 mL of 1 M nitric acid solution resulted in the lowest acidity of the reaction solution, indicating that the addition of more oyster shells is economically inefficient (Additional file 1: Fig. S4). Strain AK13 precipitated calcium carbonate in B4O medium using oyster shell-derived calcium ions (OC). AK13 cells were visualized with minerals precipitated around them through FE-SEM (Fig. 4a). The minerals were identified as calcium carbonate by EDX analysis, which had clear carbon, oxygen, and calcium peaks (Fig. 4b). The vaterite was further confirmed as a crystalline form of precipitated calcium carbonate by XRD analysis (Fig. 4c). AK13 precipitated an average of 0.29 g of calcium carbonate with the OC separated from 1 g of oyster shell, which is about 5.09 times more than that precipitated from the same mass of calcium lactate (Fig. 4d). This data suggests that oyster shells are not inadequate in terms of calcium ion content compared to the mineral precursor used previously in bio-concrete production (Jonkers et al. 2010).

### Measurement of mortar compressive strengths

**Fig. 5:**
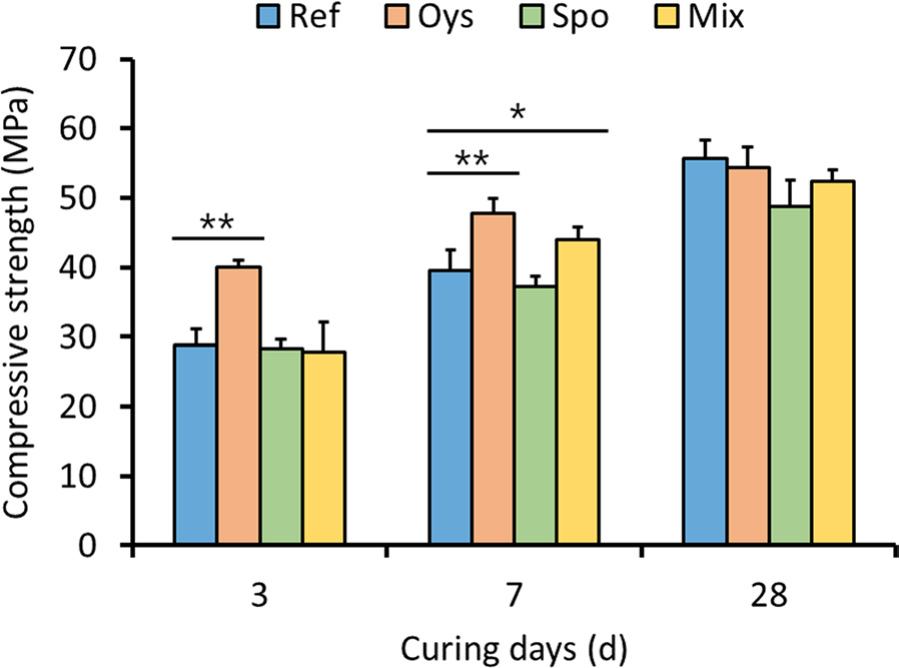
Compressive strength development of four types of mortar specimens per curing period. *p < 0.01; **p < 0.001

Calcium ions isolated from oyster shells were mixed into the mortar to measure the effect on the compressive strength, which is one of the most important properties of concrete. Bacterial spores were produced in EM and collected by centrifugation to obtain about 2.23 × 10^10^ spores per 1 g of dry weight. The OC-included mortar (Oys) exhibited a significantly higher initial compressive strength on days 3 and 7 than the reference (Fig. 5). The compressive strength of the Oys mortar on the 3 and 7 days was 74% and 87% of those on the 28 day, respectively. Conversely, the compressive strength of the reference mortar specimen (Ref) on days 3 and 7 was 53% and 71%, respectively. The mortar containing AK13 spores (Spo) had a trend of insufficient compressive strength development over time. The compressive strength at days 7 and 28 of the Spo mortar was 94.4% and 94.2% of the Ref compressive strength, respectively. However, the Mix mortar, which contained both OC and spores, showed compensation in a decrease in compressive strength at days 7 and 28 compared to the Spo mortar. The Mix mortar also exhibited a slightly increased compressive strength on the 7 day compared to that of the Ref specimen. The compressive strength of days 3 and 7, compared to day 28 of the Mix mortar was 54% and 84%, respectively. These data suggest that the strength development rate of Mix on day 3 was decelerated compared to that of Oys, likely due to the incorporation of spores and/or EM residues.

### Effect of OC-soybean meal-solution on the crack-sealing of cement mortar

Another screen of six agricultural by-products was conducted, this time to identify a substrate that promotes the germination, growth, and metabolic activities of AK13 and thus promote MICP inside the concrete matrix. While SM was used as a spore-forming substrate in the composition of EM (Figs. 1a and 3) it failed to efficiently germinate AK13 spores, possibly because of an insufficient lysine content (Additional file 1: Fig. S5b) (Ravindran 1992). Soybean meal was selected as the highest growth and germination rates for AK13 vegetative cells and spores were recorded in soybean meal-broth and plate (Additional file 1: Fig. S5a, b). Therefore, a soybean meal-derived nutrient solution (SN) was applied in a subsequent crack-sealing experiment to the cracks in each experimental mortar. Soybean meal replaced yeast extract that is mainly used in bio-concrete research (Seifan et al. 2016; Vijay et al. 2017). The SN was set to pH 10 with 2 M NaOH before using to maintain the alkalinity of the mortar specimens.

**Fig. 6:**
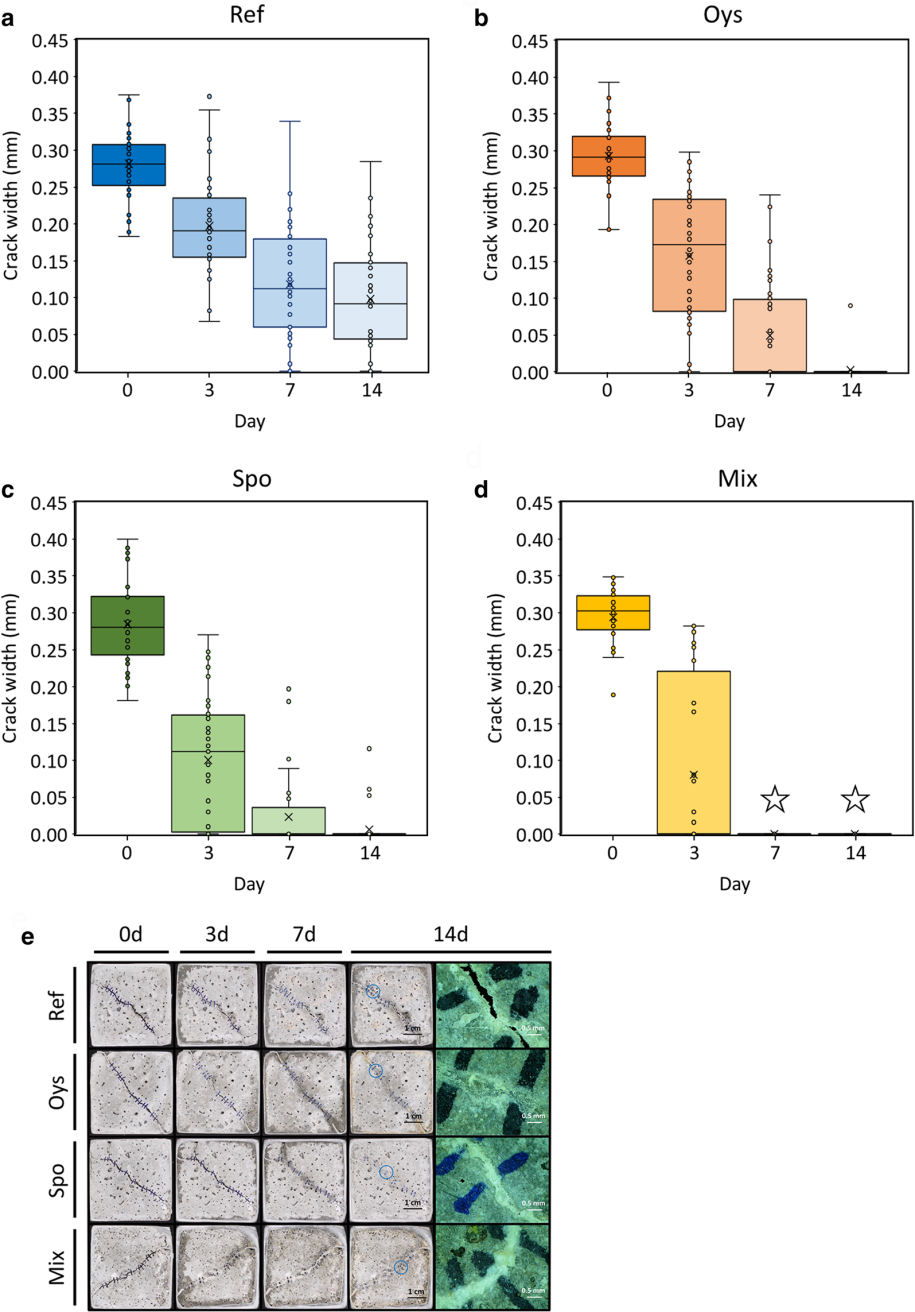
Measurement of crack-healing rates of mortar specimens treated with SN. **a**–**d** The decreasing crack width in specimens treated with SN once a day. An asterisk (☆) indicates cracks that were healed completely. **e** Progression of calcium carbonate precipitation in cracks with daily SN treatment

In all four mortar specimens, the crack sealed more rapidly following SN administration (Fig. 6a–d) compared to the control group (Additional file 1: Fig. S6a–d). The change in crack-sealing rates from SN application was at least 1.36 and, at most, 2.1-fold in the Ref and Mix mortars, respectively, as a result of comparing the slopes of the linear trend lines against the changes in crack widths over time (Additional file 1: Table S3). The increases in crack-sealing rates by SN were greatest for the Mix mortar and decreased in the following order: Spo, Oys, and Ref. Crack-sealings occurred even in the control group without administration of SN (Additional file 1: Fig. S6a–d). The stereomicroscope images showed calcium carbonate formation even on the crack of the untreated control mortar (Additional file 1: Fig. S6e) and the SN-untreated Mix mortar had improved crack-sealing compared to other SN-untreated mortar types (Fig. 6d and Additional file 1: Fig. S6d). The highest crack-closure rate overall was observed in the Mix mortar at 2.19 times that of the Ref (Additional file 1: Table S3). Additionally, calcium carbonate crystals precipitated at least slightly in the cracks of all mortar specimens, but the largest crystal-formation area was observed in the Mix mortar that benefited from OC, SN, and bacterial spores (Fig. 6e). Both OC and SN promoted the crack-sealing of cement mortar, especially for the Mix mortar.

**Fig. 7:**
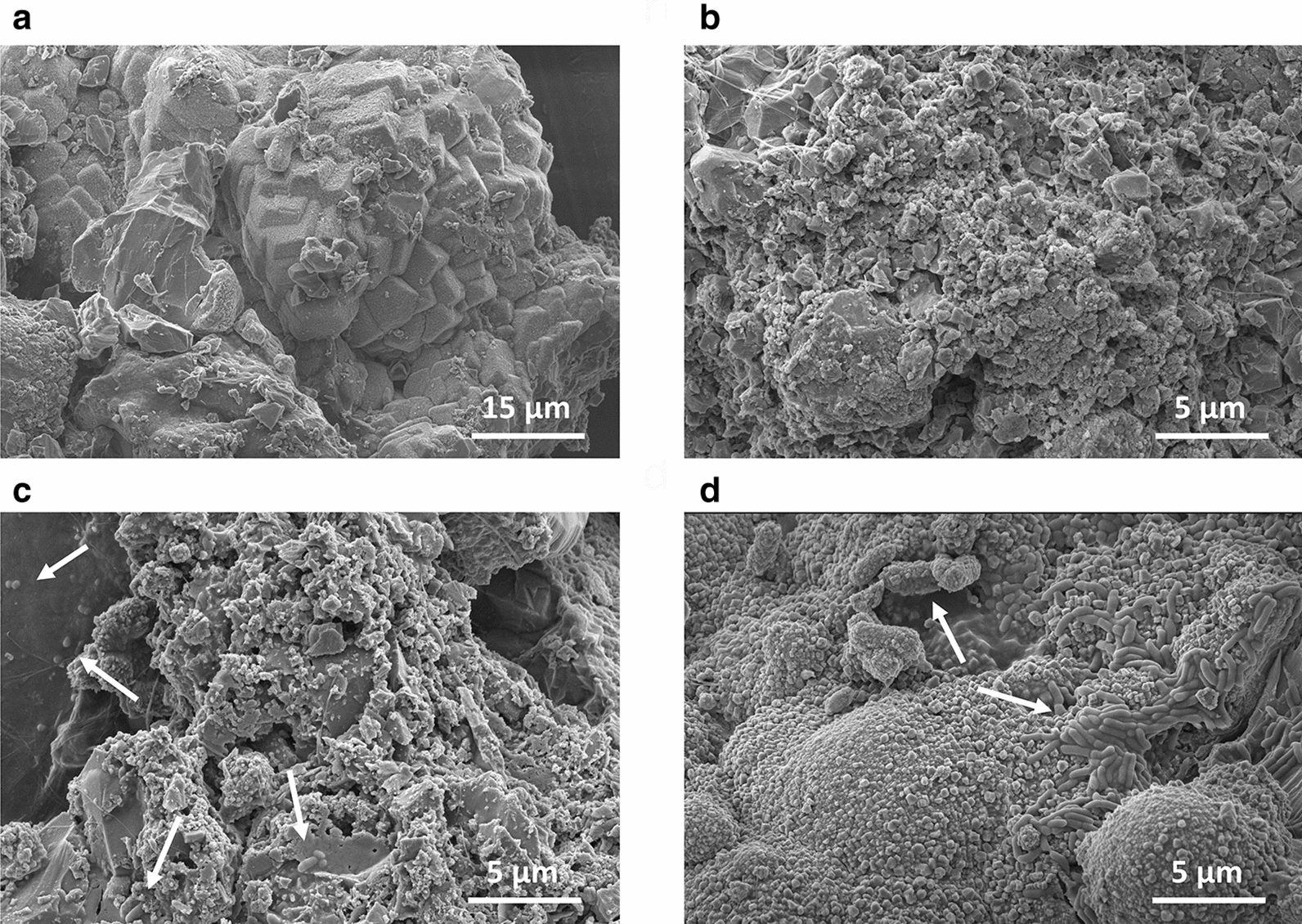
FE-SEM image of calcium carbonate precipitated in the cracks of SN-treated specimens. **a**, **b** Images of calcium carbonate formed in the cracks of Ref and Oys mortars on day 14 post-crack induction. **c**, **d** Calcium carbonate and bacteria collected from the cracks of Spo and Mix mortars treated daily with SN for 14 days post-crack induction. Arrows point to vegetative cells or spores

FE-SEM imaging was performed on each of the calcium carbonate precipitates in the 14 day cracks of the four mortar specimens shown in Fig. 6e. Morphological differences were observed in the Mix mortar precipitated calcium carbonates compared to the rest (Fig. 7a–d). Calcium carbonates precipitated in an amorphous or round shape like those in the B4O medium (Fig. 4a) in OC-containing Oys mortar and Spo mortar whose cracks were sealed by MICP of strain AK13 (Fig. 7b, c). These calcium carbonates were presumed to be vaterite, like those from B4O medium precipitated using OC by non-ureolytical MICP of strain AK13 (Fig. 4a). The overall appearance of calcium carbonate precipitates in the Ref specimen also looked like those in the Oys and Spo mortars (Fig. 7a). The precipitation of calcium carbonates in Ref and Oys mortars was presumed to be due to natural carbonation (Beuvier et al. 2011; Wiktor and Jonkers 2011). The number of observed bacteria also differed between Mix and the other mortar types. Vegetative cells and spores were sparsely visible in the Spo mortar, but prolific in the Mix mortar (Fig. 7c, d). Only a small number of bacteria, which appeared to be curing water contaminants, were observed in the Ref mortar, and no bacteria were observed in the Oys mortar (Additional file 1: Fig. S7a).

## Discussion

Sesame meal (SM) was selected among six agricultural by-products as a spore-forming substrate equivalent to Difco™ sporulation (DS) medium (Fig. 1a). High amounts of AK13 spore formation in SM-medium might be explained by the high calcium and relatively low other nutritional contents in SM (Ravindran 1992). In SM-medium, AK13 entered the death phase within 24 h, likely due to low nutrients (Additional file 1: Figs. S3a and S5a), which increased the starvation stress and promoted sporulation. The relatively high sporulation rate of AK13 in SM led to its selection as a growth and sporulation factor. The advantages of spores are to give validity to the applications of spore-forming bacteria such as *Bacillus* spp., *Lysinibacillus* spp., and *Sporosarcina* spp. to the bio-concrete in other researches (Jonkers and Schlangen 2007; Jonkers et al. 2010; Seifan et al. 2016; Vijay et al. 2017; Zhang et al. 2017; Peplow 2020). Bacterial spores are more resistant to heat and drying than vegetative cells. These properties may enable spores to better withstand the harsh conditions of concrete, including the heat of hydration that occurs inside curing concrete and the dry concrete matrix (Jang et al. 2020). In addition, bacteria can be spray-dried for convenience in storage and transport, but vegetative cell will not be able to withstand the high-temperature during vaporization process (Hong et al. 2019; Jang et al. 2020; Jung et al. 2020; Ryu et al. 2020).

NaCl was selected as a growth-promoting factor for AK13. The physiological functions of alkaliphilic *Bacillus* spp. such as growth, sporulation, germination, and motility are all enhanced in the presence of sodium ions (Horikoshi 2011). Strain AK13 increased the expression of Mrp antiporter-related genes under alkaline conditions (Fig. 1b), which suggest that the sodium ion-based adaptation mechanism (Additional file 1: Fig. S2) is also important for AK13 growth in alkaline conditions. Strain AK13 also up-regulated the expression of the ECF transporter-related genes at pH 10 (Fig. 1c). The ECF transporter mediates the uptake of essential micronutrients such as riboflavin, thiamine, biotin, and tryptophan (Slotboom 2014). Riboflavin, a precursor of flavin mononucleotide (FMN) and flavin adenine dinucleotide (FAD), is the most important organic cofactor in the electron transport system (Sepúlveda-Cisternas et al. 2018). It is possible that riboflavin may support the respiratory process of AK13 in alkaline conditions (Additional file 1: Fig. S2).

Higher spore formation of AK13 was induced in economical medium (EM; consisted of SM, NaCl, and riboflavin) than in DS medium. AK13 spore production in EM is efficient but may not be sufficient. Strain AK13 capable of forming spores (7.56 × 10^7^ spores/mL) may have inferior spore-forming ability compared to other bacteria such as *B. subtilis* or *L. sphaericus*, which can form 10^10^ or more spores/mL (Sasaki et al 1998; Chen et al. 2010). However, AK13 is highly resistant to alkaline conditions as high as pH 12 (Lee and Park 2019; Jung et al. 2020; Shin et al. 2020). The alkali tolerance of AK13 might allow crack-healing to begin early, even if the initial alkalinity of the mortar, about pH 12, is slightly decreased by influent water. Early crack-healing or crack-sealing may suppress excessive fissure expansion. Strain AK13 may also have positive effects when co-applied with other bacteria such as providing alkali resistance to the partner strain, improving biofilm formation, and improving precipitated calcium carbonate, even in small quantities (Lee and Park 2019). Therefore, the relatively lower spore production may be overcome by other advantages of strain AK13.

Additional mineral precursors should be added to accelerate crack-healing of bio-concrete (Jonkers et al. 2010). In this study, oyster shells, a by-product of the oyster-farming industry, were used as an alternative calcium source, but not sufficient enough to replace major calcium sources such as calcium lactate, calcium acetate, which might be also carbon sources for bacterial growth. A pretreatment was required to dissociate calcium ions from the oyster shell with nitric acid due to the low solubility of calcium carbonate (15 mg/L at 25 °C in water) (Wang et al. 2014). Other acids, e.g. hydrochloric acid, sulfuric acid, and acetic acid, etc. could also be used to decompose calcium carbonate, but nitric acid was selected in consideration of the effects of other anions (Cl^−^, SO_4_^2−^, CH_3_COO^−^) on the physical properties of mortar or concrete. Calcium chloride, obtained by treating oyster shells with hydrochloric acid, can accelerate the setting and hardening of concrete but its chloride ions can also corrode reinforcing steel inside the matrix (Montemor et al. 2003; Myrdal 2007). Sulfate, a hydration product of sulfuric acid, can react with the calcium hydroxide and calcium aluminate present in a concrete matrix to form secondary ettringite and gypsum, which can lead to expansion, cracking, and strength reduction (Panesar 2019). Calcium acetate had a positive effect on Brazilian splitting tensile strength and the uniaxial compressive strength of mortar (Zhang et al. 2015). However, the compressive strength 28 days later was 76.92% compared to the reference in another study (Jonkers et al. 2010). Acetic acid is also a weak acid and has an irritating odor, thereby excluding it from use. In contrast, calcium nitrate, such as the main component of the reactant of oyster shell and nitric acid, shortens the hardening time (like calcium chloride) but does not negatively affect the properties of concrete. Calcium nitrate can also prevent chloride-derived corrosion of steel reinforcement (El-Reedy 2017). Additionally, nitrate can function as an electron acceptor for nitrate-reducing bacteria, thereby allowing self-healing to occur even in the hypoxic environments that are deep in the concrete matrix and flooded areas (Erşan et al. 2016). Therefore, the oyster shell was treated with nitric acid to form calcium ions with nitrates, which are harmless to structure and could have a positive effect on properties of concrete.

Vaterite, such as that surrounding AK13 in the B4O medium (Fig. 4a), is a polymorphism of calcium carbonate like calcite and aragonite. Calcite is the most stable form under normal atmospheric pressure with room temperature, while vaterite and aragonite are metastable and easily transform into calcite-form calcium carbonate in response to water or high temperature (Han et al. 2006). Vaterite can be formed by mixing calcium salt with carbonate-ion solution, which is the same mechanism as calcium carbonate precipitation through AK13 respiration (Fig. 4a) (Beuvier et al. 2011). When sufficient nucleation sites are present, calcium carbonate undergoes agglomeration and recrystallization at the nanoparticle level to form spherical vaterite with a diameter of several microns (Decho 2010). The unstable, relatively soluble vaterite crystals subsequently dissolve in water and transform into calcite. Han and colleagues (2006) note that the calcium-ion concentration is a determining factor for the growth of calcite from vaterite. Only calcite was observed in aqueous solutions with an initial calcium-ion concentration of at least 0.3 mol/L (Han et al. 2006). The reason that vaterite was the primary product of AK13 MICP (Fig. 4c) may be an insufficient initial concentration of oyster shell-derived calcium ions (OC). Another possibility is that the absence of urea may have caused the predominant precipitation of vaterite. Urea hydrolysis is required for bacterial precipitation of calcite-form calcium carbonate (Stocks-Fischer et al. 1999). Urea has been used as a substrate for efficient calcium carbonate precipitation in bio-concrete, especially with *L. sphaericus* and *S. pasteurii* (Seifan et al. 2016; Vijay et al. 2017). However, urea hydrolysis forms ammonia, which negatively affects humans and buildings as well as being toxic to aquatic organisms (Erşan et al. 2016). Urea hydrolysis may lead to an excessive nitrogen load by generating two ammonium ions for each carbonate (Jonkers et al. 2010). Without urea, other bacterial, including strain AK13, metabolic activities like respiration can still increase the concentration of carbonates in aqueous solutions. The high carbonate concentration then enables non-ureolytic calcium carbonate precipitation by binding with calcium ions derived from cementitious materials like calcium hydroxide or additional calcium sources (Dupraz et al. 2009; Decho 2010; Wiktor and Jonkers 2011; Lee et al. 2017). AK13 cells can produce carbonate through aerobic respiration and precipitates biologically-derived calcium carbonate (Jung et al. 2020). Optimization of the OC input should be performed to facilitate calcite precipitation by AK13.

The compressive strengths of Oys and Mix mortars on 3 and 7 days were significantly increased compared to those of Ref mortar. The remaining calcium ions and nitrates following nitric-acid treatment of oyster shell powder seemed to increase the initial compressive strengths of Oys and Mix mortars (Myrdal 2007). Similarly, even when non acid-treated oyster shells are reacted with cementitious materials, the initial compressive-strength development is accelerated, possibly due to absorption of water by the oyster-shell-aggregate, which dropped the water-cement ratio (Yang et al. 2005). In contrast, a decrease in long-term compressive strength could be caused by the replacement of fine concrete aggregates with ground oyster shells (Yang et al. 2010). Additional experiments are required to understand the effect of acid-treated oyster shell on the long-term strength development of concrete.

Decades of bio-concrete research have concluded that when bacteria in cement mortar precipitate calcite-form calcium carbonate, the compressive strength increases due to the reduction of permeability and porosity of mortar (Vijay et al. 2017). However, the addition of bacteria reduced the compressive strength of the Spo mortar, which may be due to the presence of organic matter such as bacterial cells and SM-residues from EM (Fig. 5). Organic matter impedes the hydration of cement particles, thereby reducing strength development (Jonkers et al 2010; Wang et al. 2014; Erşan et al. 2015; Jang et al. 2020). Nitrate-extracted OC is expected to be a cost-effective admixture to bio-concrete that compensates for the organic matter-induced decrease in compressive strength.

Among the six agricultural by-products, soybean meal was most effective in germination and growth of AK13 spores, and thus was used as a nutrient to replace the yeast extract that has been used in decades of bio-concrete research (Seifan et al. 2016; Vijay et al. 2017). Soybean meal is a major protein source that supplies amino acids including lysine, methionine, and threonine, which could explain the relatively high spore-germination of AK13 (Willis 2003). The concentration of soybean meal (SN) was diluted to half of that used previous screening experiment (Additional file 1: Fig. S5a) before being introduced into the cracks of each mortar specimen. The rationale for the dilution was because calcium ions or carbonates can be sequestered in the presence of excessive organic matters with negatively-charged functional groups and possibly inhibit calcium carbonate precipitation (Decho 2010). The increase in crack-sealing rate due to SN treatment was particularly evident in Mix and Spo mortars (Additional file 1: Table S3), which suggest an increase in the bacterial metabolic rates due to the addition of nutrients, which resulted in the increased MICP in the cracks of Mix and Spo mortars.

Calcium ions extracted from oyster shells had a positive effect on the preservation of bacteria and crack-sealing. The calcite-formed calcium carbonates were precipitated in the Mix mortar likely due to the addition of OC (Fig. 7d). OC seemed to facilitate calcite formation in the Mix mortar cracks (Han et al. 2006). The calcium carbonate precipitation observed in the Mix mortar was positively correlated with bacterial counts (Figs. 6d, 7d, and Additional file 1: Fig. S7d), suggesting that OC significantly contributed to the survival of bacteria and MICP-induced crack-sealing, along with SN. In the Mix mortar, the bacteria were abundant and observed in solidified states completely covered with minerals (Fig. 7d, Additional file 1: Fig. S7c, d). Bacteria were only abundant in the Mix mortar, which might be explained by an increase in biofilm formation by OC addition because calcium ions can stabilize and strengthen biofilms (Das et al. 2014). Calcium ions can also increase bacterial EPS secretion, potentially explaining the mucous membrane-like texture of the calcium carbonate that originated from the cracks of the Mix mortar (Additional file 1: Fig. S7d) (Patrauchan et al. 2005). These factors, along with EPS-related nucleation site enrichment, likely contributed to the observation that the fastest crack-sealing and most robust form of precipitated calcium carbonate were observed in the Mix mortar (Decho 2010). Experimental data indicates that the addition of OC to yeast extract-medium increased biofilm formation, supporting the hypothesis that calcium ions strengthened the biofilm (Additional file 1: Fig. S8). These data suggest a reason for the small number of bacteria or only their traces (Additional file 1: Fig. S7b) observed in the Spo mortar—the lack of a calcium-strengthen biofilm allowed cell loss during the ethanol wash prior to FE-SEM imaging. The bacteria in the Mix mortar may have been better preserved contrary to those of Spo mortar from the voltexing in ethanol, thanks to the fossilization, i.e. formation of the thick calcium carbonate layer around the cell wall, along with the protection from the richly formed biofilm (Fig. 7d, Additional file 1: Fig. S7c, d). Therefore, the addition of calcium ions to bio-concrete production is a logical choice, and oyster shells are an ideal source of cost-effective calcium to increase bacterial survival and promote crack-sealing.

## Supplementary Information

**Additional file 1: Table S1.** Selected three factors and their levels for central composite design. **Table S2.** Experimental design in the optimization to form an economical medium. **Table S3.** Equations of the linear trend lines of changes in crack width over sealing periods in four mortar specimens. **Figure S1.** Phenotypic and gene-expression comparison of strain AK13 in pH 7 and 10 LB media. (a) Growth curves of the strain AK13 in each of the above two conditions. Arrows indicate when total RNAs were extracted. (b) Organization by COG category of the genes whose expressions were up-regulated under neutral or alkaline conditions. **Figure S2.** The adaptation mechanisms of alkaliphilic *Bacillus* spp. to the alkaline environment including the function of riboflavin in the respiratory chain. **Figure S3.** Comparison of growth and spore formation when NaCl or riboflavin was added to sesame meal-medium. (a) Comparison of growth when each of two growth promoting factors were added to sesame meal-medium. (b) Changes in spore formation when each of NaCl or riboflavin were included in sesame meal-medium. The concentrations of sesame meal, NaCl, and riboflavin were 10 g, 10 g, and 10^–4^ g per liter, respectively. ND: not detected. *: p < 0.05; **: p < 0.01. **Figure S4.** Changes in acidity of reactants when different amounts of oyster shells were added in 1 M of nitric acid solution. **Figure S5.** Screening of agricultural by-products to produce nutrient solution for promoting bacterial growth, metabolism, and crack-sealing. (a) Growth of strain AK13 from the supernatants after centrifugations of each autoclaved agricultural by-product solutions. (b) Screening of agricultural by-products that showing the maximum germination rate for purely isolated spores of strain AK13. **Figure S6.** Measurement of crack-sealing rates of mortar specimens without SN treatment. (a–d) Changes in crack width of control specimens dispensed with DW instead of nutrient solution. (e) Images of mortar cracks over time when DW is administered every day. **Figure S7.** Additional FE-SEM images of calcium carbonates precipitated in the cracks of the mortar specimens. (a) Calcium carbonate derived from cracks in the Ref mortar, and the presumed form of bacteria. (b) Holes found in calcium carbonate obtained from cracks in the Spo mortar. (c, d) Numerous bacteria and mucosa-textured surfaces found in calcium carbonate precipitated in the cracks of the Mix mortar. Arrows in d indicate areas where large amounts of bacteria were buried in what appears to be biofilm or mineral layers. **Figure S8.** Visualization of differences in biofilm formation without and with addition of OC using confocal laser scanning microscope (CLSM). YE and OC were added at a concentration of 0.4% and 1.0%, respectively. Preparation for CLSM imaging was conducted in the same manner as in the previous study (Lee and Park 2019). YE: yeast extract.

## Data Availability

All data generated and analyzed during this study are included in this published article.
