# Supplementary material for: Agricultural by-products and oyster shell as alternative nutrient sources for microbial sealing of early age cracks in mortar
Source: AMB Express. 2021 Jan 6;11:11. doi: 10.1186/s13568-020-01166-5 (PMC7788133; doi:10.1186/s13568-020-01166-5)
Supplement: Supplementary file 1 — Additional file 1: Table S1. Selected three factors and their levels for central composite design. Table S2. Experimental design in the optimization to form an economical medium. Table S3. Equations of the linear trend lines of changes in crack width over sealing periods in four mortar specimens. Figure S1. Phenotypic and gene-expression comparison of strain AK13 in pH 7 and 10 LB media. (a) Growth curves of the strain AK13 in each of the above two conditions. Arrows indicate when total RNAs were extracted. (b) Organization by COG category of the genes whose expressions were up-regulated under neutral or alkaline conditions. Figure S2. The adaptation mechanisms of alkaliphilic Bacillus spp. to the alkaline environment including the function of riboflavin in the respiratory chain. Figure S3. Comparison of growth and spore formation when NaCl or riboflavin was added to sesame meal-medium. (a) Comparison of growth when each of two growth promoting factors were added to sesame meal-medium. (b) Changes in spore formation when each of NaCl or riboflavin were included in sesame meal-medium. The concentrations of sesame meal, NaCl, and riboflavin were 10 g, 10 g, and 10–4 g per liter, respectively. ND: not detected. *: p < 0.05; **: p < 0.01. Figure S4. Changes in acidity of reactants when different amounts of oyster shells were added in 1 M of nitric acid solution. Figure S5. Screening of agricultural by-products to produce nutrient solution for promoting bacterial growth, metabolism, and crack-sealing. (a) Growth of strain AK13 from the supernatants after centrifugations of each autoclaved agricultural by-product solutions. (b) Screening of agricultural by-products that showing the maximum germination rate for purely isolated spores of strain AK13. Figure S6. Measurement of crack-sealing rates of mortar specimens without SN treatment. (a–d) Changes in crack width of control specimens dispensed with DW instead of nutrient solution. (e) Images of mortar cracks over time whe [file 13568_2020_1166_MOESM1_ESM.pdf]

Journal: AMB Express

Title: Agricultural by-products and oyster shell as alternative nutrient sources for microbial sealing of early age cracks in mortar

Minyoung Hong[1], Indong Jang[2], Yongjun Son[1], Chongku Yi[2] and Woojun Park[1*]

[1]Laboratory of Molecular Environmental Microbiology, Department of Environmental Science and Ecological Engineering, Korea University, Seoul 02841, Republic of Korea

[2]Construction Management and Material Laboratory, School of Civil, Environmental and Architectural Engineering, Korea University, Seoul 02841, Republic of Korea

[*]**Corresponding author:** Dr. Woojun Park, Department of Environmental Science and Ecological Engineering, Korea University, Seoul 02841, Republic of Korea

E-mail: wpark@korea.ac.kr

Fax: +82-2-953-0737

Phone: +82-2-3290-3067

Table S1. Selected three factors and their levels for central composite design.

Factor	Symbol	Coded level				
		-α	-1	0	+1	+α
Sesame meal (%, w/v)	X_1	0.16	0.50	1.00	1.50	1.84
NaCl (%, w/v)	X_2	0.13	0.40	0.80	1.20	1.47
Riboflavin (%, 10^{-4} w/v)	X_3	0.22	1.12	2.44	3.76	4.65

Table S2. Experimental design in the optimization to form an economical medium.

Standard order	Factors						Response
	Sesame meal (%, w/v)	Coded level	NaCl (%, w/v)	Coded level	Riboflavin (%, 10^{-4} w/v)	Coded level	Spores (log CFU/ml)
1	0.50	-1	1.00	-1	1.12	-1	7.07
2	1.50	+1	1.00	-1	1.12	-1	5.89
3	0.50	-1	2.00	+1	1.12	-1	7.32
4	1.50	+1	2.00	+1	1.12	-1	6.47
5	0.50	-1	1.00	-1	3.76	+1	6.65
6	1.50	+1	1.00	-1	3.76	+1	6.07
7	0.50	-1	2.00	+1	3.76	+1	6.9
8	1.50	+1	2.00	+1	3.76	+1	6.78
9	0.16	-α	1.50	0	2.44	0	7.36
10	1.84	+α	1.50	0	2.44	0	5.96
11	1.00	0	0.66	-α	2.44	0	6.65
12	1.00	0	2.34	+α	2.44	0	6.95
13	1.00	0	1.50	0	0.22	-α	6.97
14	1.00	0	1.50	0	4.65	+α	6.44
15	1.00	0	1.50	0	2.44	0	7.08

16	1.00	0	1.50	0	2.44	0	7.34
17	1.00	0	1.50	0	2.44	0	7.32
18	1.00	0	1.50	0	2.44	0	7.5
19	1.00	0	1.50	0	2.44	0	7.29
20	1.00	0	1.50	0	2.44	0	7.21

Table S3. Equations of the linear trend lines of changes in crack width over sealing periods in four mortar specimens.

Nutrient	Type of specimens	Linear trend line	Coefficient of determination (%)
(-)	Ref	Y = -0.0464x + 0.321	0.87
	Oys	Y = -0.0593x + 0.3351	0.95
	Spo	Y = -0.0704x + 0.3534	0.95
	Mix	Y = -0.1016x + 0.3995	0.95
(+)	Ref	Y = -0.0631x + 0.3313	0.94
	Oys	Y = -0.098x + 0.3704	0.96
	Spo	Y = -0.1304x + 0.3967	0.95
	Mix	Y = -0.2135x + 0.5076	0.99

Fig. S1. Phenotypic and gene-expression comparison of strain AK13 in pH 7 and 10 LB media. (a) Growth curves of the strain AK13 in each of the above two conditions. Arrows indicate when total RNAs were extracted. (b) Organization by COG category of the genes whose expressions were up-regulated under neutral or alkaline conditions.

a

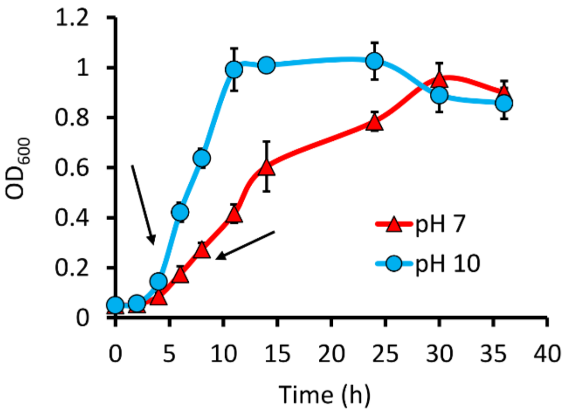

b

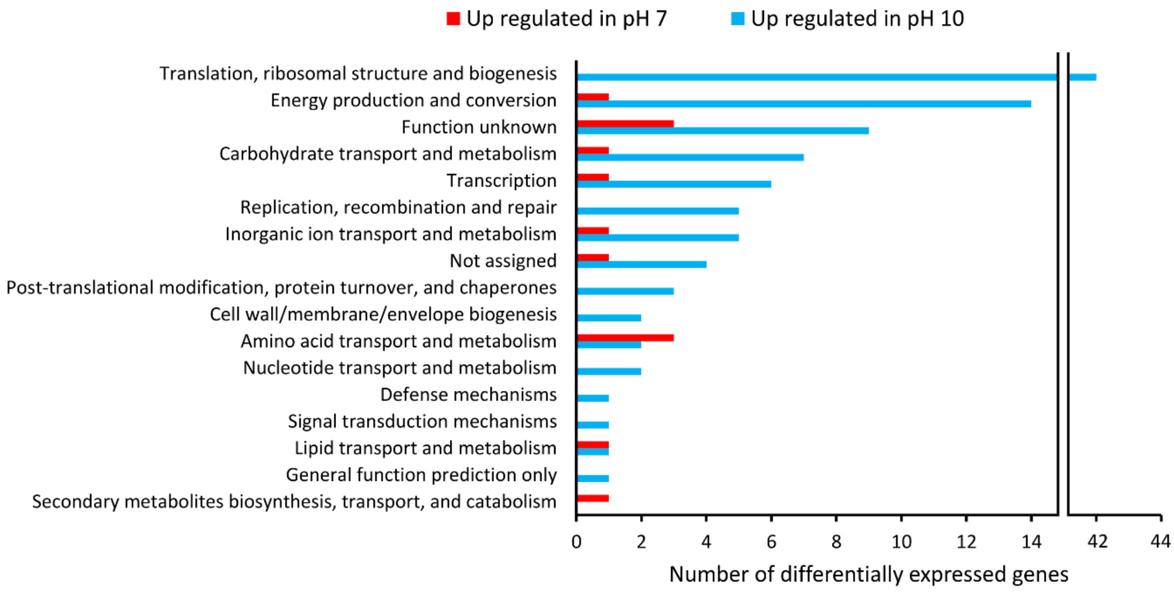

Fig. S2. The adaptation mechanisms of alkaliphilic *Bacillus* spp. to the alkaline environment including the function of riboflavin in the respiratory chain.

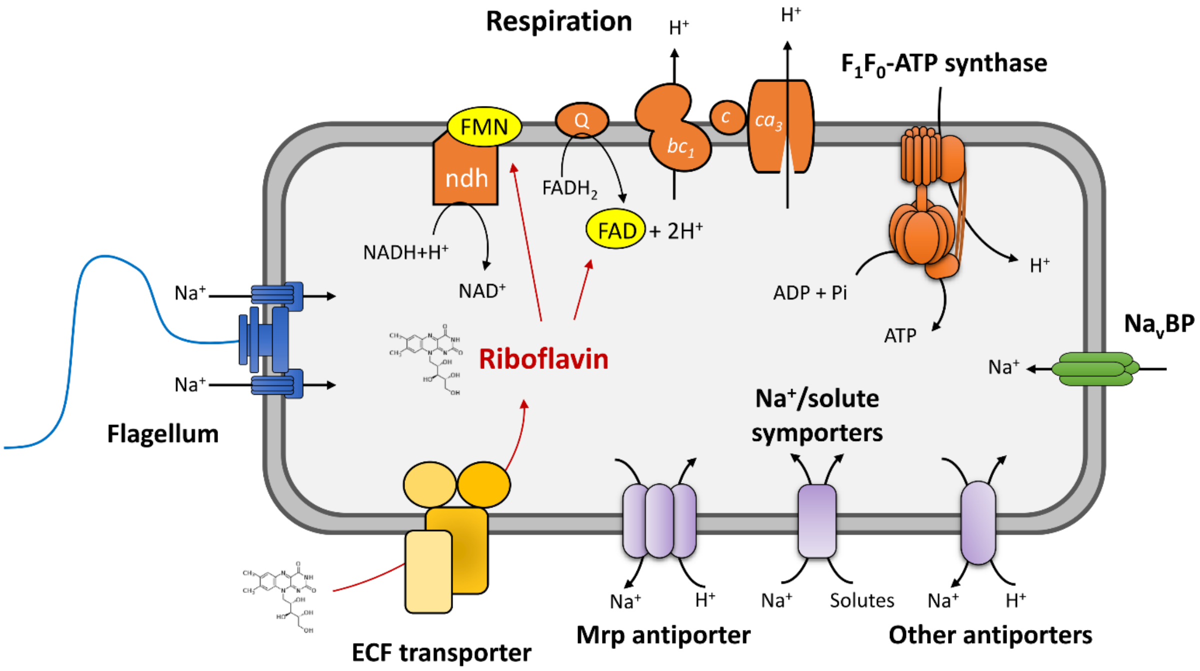

Fig. S3. Comparison of growth and spore formation when NaCl or riboflavin was added to sesame meal-medium. (a) Comparison of growth when each of two growth promoting factors were added to sesame meal-medium. (b) Changes in spore formation when each of NaCl or riboflavin were included in sesame meal-medium. The concentrations of sesame meal, NaCl, and riboflavin were 10 g, 10 g, and 10^{-4} g per liter, respectively.

ND : not detected. *: $p < 0.05$; **: $p<0.01$.

a

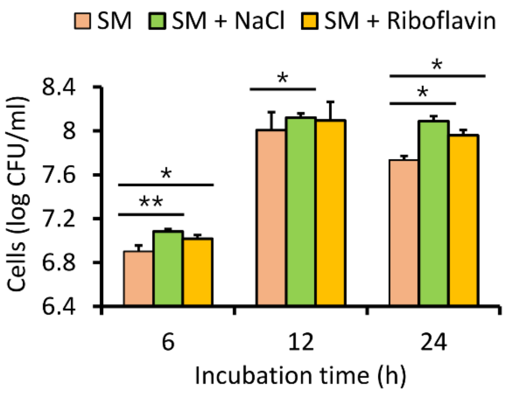

b

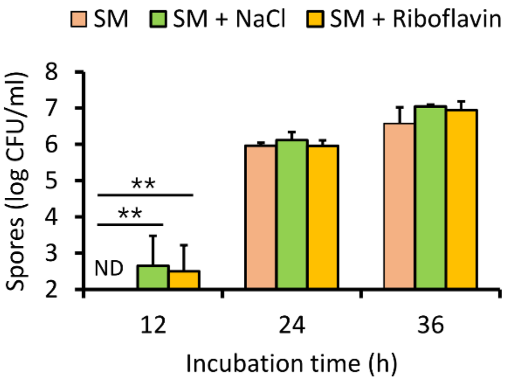

Fig. S4. Changes in acidity of reactants when different amounts of oyster shells were added in 1M of nitric acid solution.

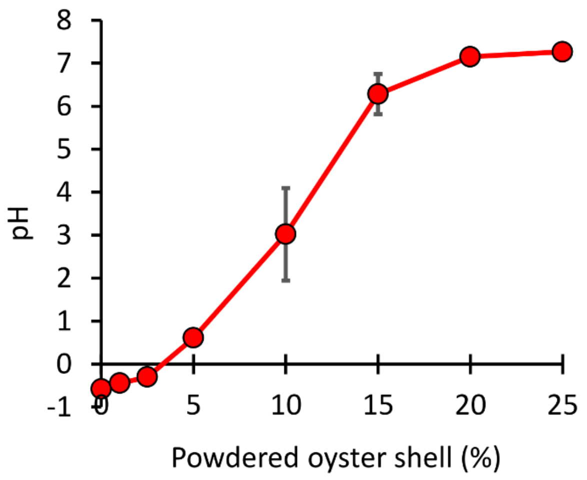

Fig. S5. Screening of agricultural by-products to produce nutrient solution for promoting bacterial growth, metabolism, and crack-sealing. (a) Growth of strain AK13 from the supernatants after centrifugations of each autoclaved agricultural by-product solutions. (b) Screening of agricultural by-products that showing the maximum germination rate for purely isolated spores of strain AK13.

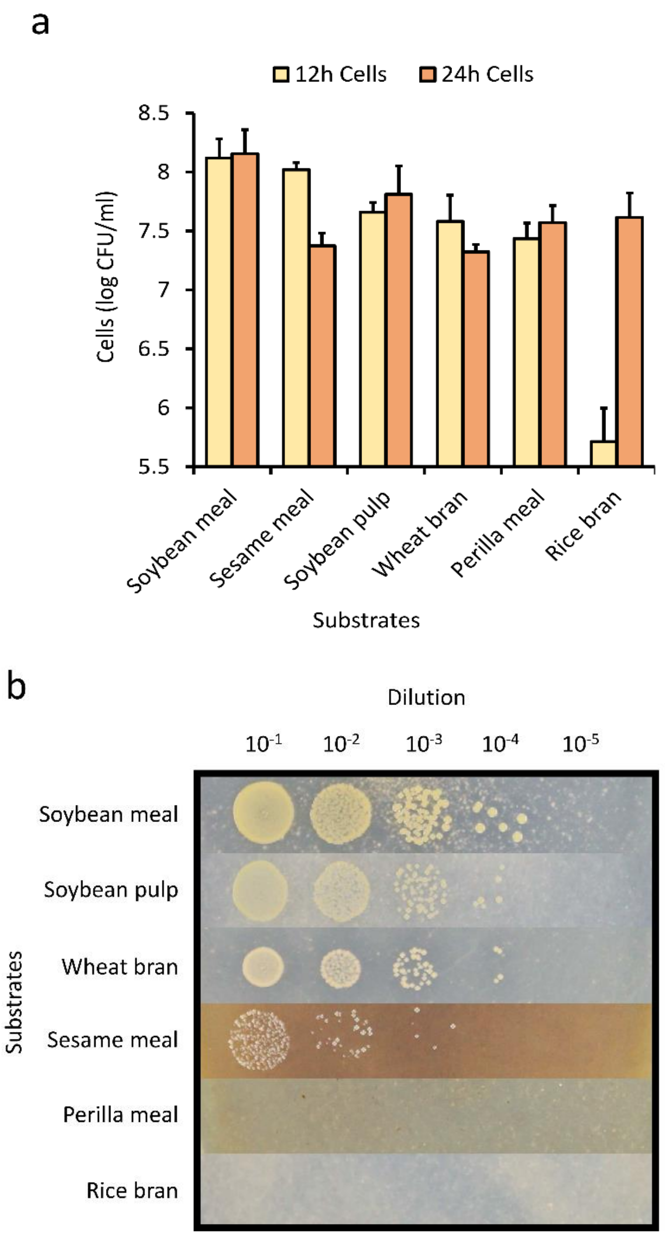

Fig. S6. Measurement of crack-sealing rates of mortar specimens without SN treatment. (a – d) Changes in crack width of control specimens dispensed with DW instead of nutrient solution. (e) Images of mortar cracks over time when DW is administered every day.

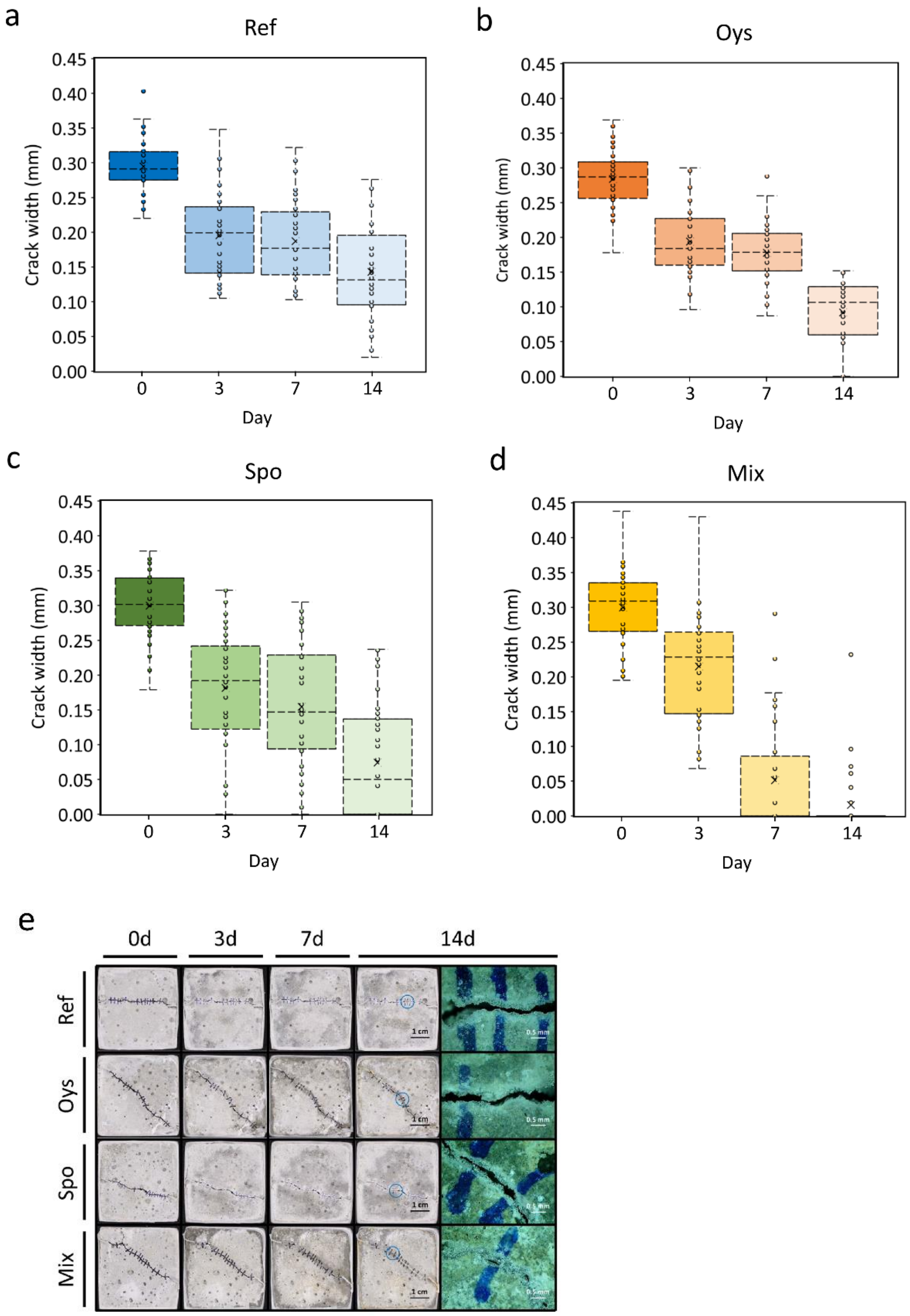

Fig. S7. Additional FE-SEM images of calcium carbonates precipitated in the cracks of the mortar specimens. (a) Calcium carbonate derived from cracks in the Ref mortar, and the presumed form of bacteria. (b) Holes found in calcium carbonate obtained from cracks in the Spo mortar. (c, d) Numerous bacteria and mucosa-textured surfaces found in calcium carbonate precipitated in the cracks of the Mix mortar. Arrows in S7d indicate areas where large amounts of bacteria were buried in what appears to be biofilm or mineral layers.

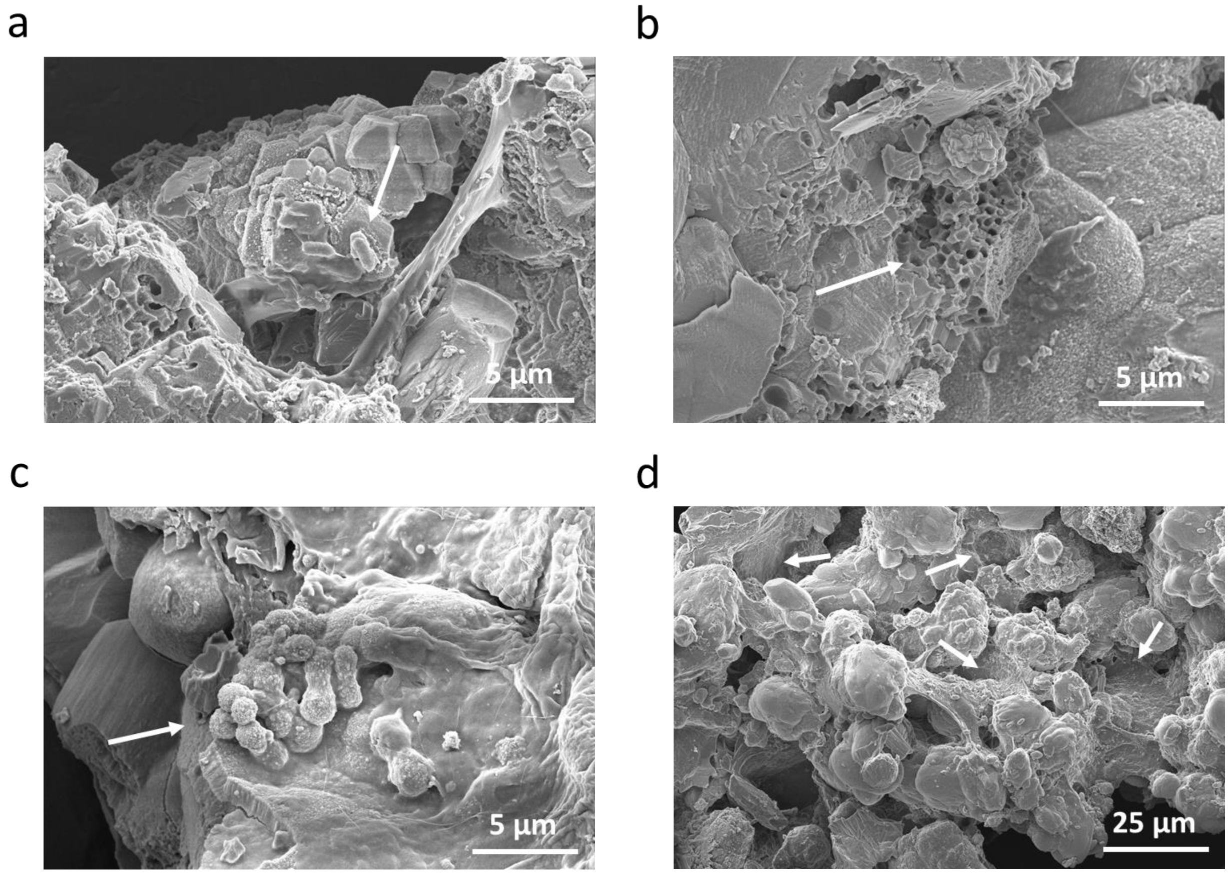

Fig. S8. Visualization of differences in biofilm formation without and with addition of OC using confocal laser scanning microscope (CLSM). YE and OC were added at a concentration of 0.4% and 1.0%, respectively. Preparation for CLSM imaging was conducted in the same manner as in the previous study (Lee and Park, 2019). YE : yeast extract.

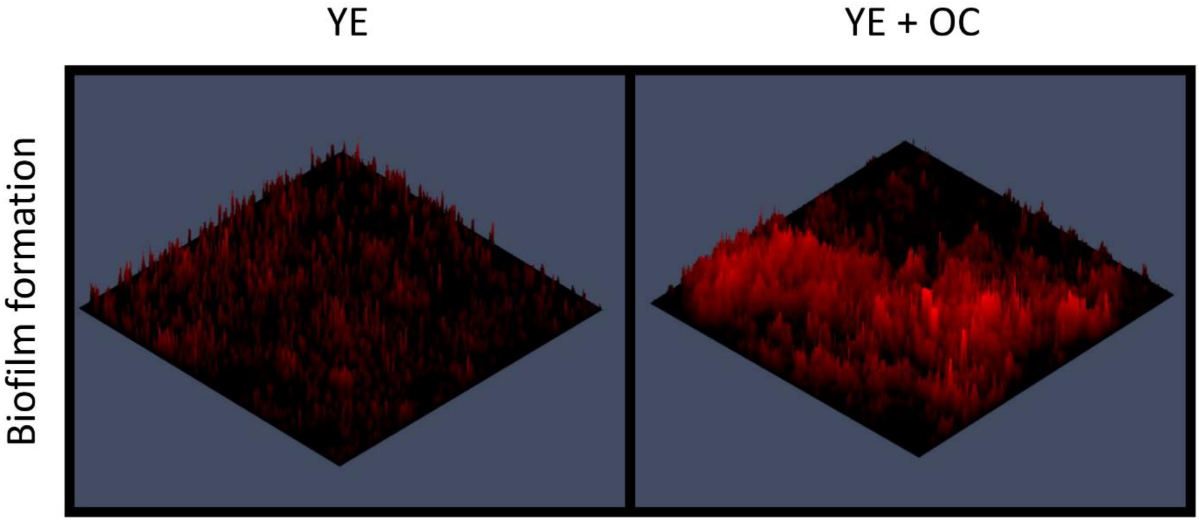

REFERENCE

1. Lee YS, Park W (2019) Enhanced calcium carbonate-biofilm complex formation by alkali-generating *Lysinibacillus boronitolerans* YS11 and alkaliphilic *Bacillus* sp. AK13. AMB Expr. 9: 49. doi.org/10.1186/s13568-019-0773-x
